# Towards Accounting for Nonhuman Agency in Technology Design for Aging

**DOI:** 10.1145/3733155.3733205

**Published:** 2025-07-17

**Authors:** Alisha Pradhan, Shaan Chopra, Pooja Upadhyay, Robin Brewer, Amanda Lazar

**Affiliations:** New Jersey Institute of Technology, Newark, New Jersey, USA; University of Washington, Seattle, USA; University of Maryland College Park, College Park, Maryland, USA; University of Michigan, Ann Arbor, Michigan, USA; University of Maryland College Park, College Park, Maryland, USA

**Keywords:** Older adults, Nonhuman agency, Posthumanism, Voice assistants

## Abstract

Till date, research on technology design to support aging has largely adopted human-centered approaches, such as user-centered and participatory design. While this body of work continues to yield insights on technologies for older individuals, recent research is beginning to question whether other ways of knowing, that are not solely “humanistic”, can yield new understandings on technologies for aging. We pick up this call, and present emerging findings from our interviews with older adults around their use of home-based intelligent voice assistants. Building upon these findings, we discuss how attuning to the nonhuman agency and the larger material world can offer new insights on technology design for aging, such as, speak to issues of technology non-use by older adults. We discuss implications of assigning responsibility and accountability beyond older adults to nonhuman actors and how it can speak to the deficit-centered narrative that often gets associated with aging.

## 1 Introduction and Background

Research on technology design for aging, till date, remains human-centered, centered around the needs and preferences of older adults, caregivers, or other social actors. This body of work has yielded rich insights for design of technology, to not only support older adults’ health and well-being, but also, meaningful activities, such as self-expression [2, 11, 30], or leisure activities [10, 13]. However, recent work in critical gerontology is beginning to question whether a sole humanistic focus can provide a holistic perspective on technologies for aging. Andrews and Duff posit that a sole focus on the ‘human subject’ alone might fail to capture the experiences and relations that involve both the human and nonhuman world, quoting how “the aging body becomes enmeshed in technological fields and networks… so almost inevitably arises as a site for the convergence of the human and the nonhuman (hence ‘more-than-human’)” [1].

Although a turn to more-than-human aspects in the context of aging is recent, understanding and designing for more-than-human perspectives has been extensively examined in the design and human-computer interaction (HCI) literature [5, 7], and is even viewed as the next research paradigm [6]. Inspired from this body of work, and by a close read of findings from prior work on aging, we can note how nonhuman entities^1^ can play a role in the experience of aging-in-place. Modifications, additions, and removal of objects in the home, such as grab bars and raised toilet seats, are key components in interventions that aim to support older adults’ activities of daily living [8]. A sense of home can emerge through practices of managing “things,” such as downsizing [16]. Objects at home become important to people [23, 27] and may have emotional attachments in terms of the meanings they carry, such as routine and the relationships with loved ones [27]. As such, attending to nonhuman entities may have much to offer in the context of technology design for aging, yet, is often unaccounted for in a systematic way in research. In this work, we examine how attuning to nonhuman entities can yield understandings on older adults’ experiences associated with use of home-based voice assistants (a commodity smart home technology that has received significant attention for supporting older adults (e.g., [17, 26]).

## 2 Methods

We conducted 30–45 minutes long, semi-structured interviews with 24 individuals (referred with PIDs, P1-P24 in section 3) between 65 to 83 years in age, who owned and used a voice assistant such as Alexa on Amazon Echo. At the time of initiating this project, our goal was to understand how older adults use voice assistants in their homes and we asked questions around older adults’ everyday use of the voice assistant, such as what they use it for, in what locations of home they use it, or how they personify the assistant. However, participants’ accounts and our initial observations when doing the interviews in individuals’ home sensitized us towards the open question of if/how nonhuman entities or material actors shaped use of the technology in their homes. Using writing as a mode of inquiry [22] we wrote descriptive memos to unpack our data. Section 3 describes a few select phenomena from our data that illustrate active participation of nonhuman and material entities in the socio-material configurations in which older adults use technology. Following our analytic approach of focusing in-depth on understanding individual phenomena where nonhuman actors played a role, our findings present in-depth analysis of a small number of cases from the data and are not intended to be generalizable. Rather, we provide these selected cases as specific examples as they yield implications in terms of design opportunities and understandings of technology for aging.

## 3 Findings

We noted the active participation of three nonhuman actors from our participants’ accounts: walls and outlets in specific locations of the home, paper-based technology manuals, and service animal or pets at home.

### 3.1 Walls and Outlets

A wall was a salient actor for P8’s voice assistant use. P8 had placed her Google Home on a shelf right outside of the kitchen. The shelf was built on a wall that is in between the kitchen and the dining room. This specific location of the Google Home, by being close to the dining room, allowed her visiting friends to use Google Assistant for timers or for asking questions, “*So when there are four of us, we sit here…and they’ll come up with a question…like, “Ask Google.”* While convenient for this social and informational purpose, the placement of the device contributes to the wall becoming a barrier when P8 wanted to use the device for timers while cooking:

P8: I do it [set timers] for food, more than anything else, I think, because it’s right there by the kitchen. Interviewer: So do you use it from your kitchen or … P8: No, I have to step out a little bit because the wall prevents it sometimes, from hearing me.

The wall, in its specific location in the house between the kitchen and dining room, acts by restricting the propagation of sound vibrations, thereby restricting the voice assistant from successfully detecting P8’s speech. P8, in turn, learned to configure her position by stepping out of the kitchen, successfully using her Google Assistant. As another instance where the layout of home affects use of voice assistants, P23 explained how the electrical outlets in the house determined the placement of the device: *“I have a living room, dining room, L type apartment so it’s [Alexa is] in the dining [room]…it [dining room] was just a convenient place for a plug, electrical outlet.”* The outlet acts by affording certain uses of Alexa in that location and at the same time, perhaps, restricts other uses. P23’s location of Alexa in a place where she spent most of her time afforded using the voice assistant for information, music, timers, alarms, and reminders, unlike others, such as P3, who asked Alexa for weather while dressing up in the morning, because of P3’s device location in the bathroom. The wall or electrical outlet are no longer passive entities whose role is to be a support structure for the house or power the technology. Rather, in unexpected ways the wall and the outlet play a role in actively shaping use of voice assistant in terms of affording and restricting certain usage and changing user behaviors (P8 moving out of kitchen to use it).

### 3.2 Technology Manuals

Paper based technology manual was a material actor whose absence contributed to disruption and frustration in using voice assistants. Consider P13 who seems to be tech-oriented and still had an “overwhelming” experience setting up his Alexa:

I ran an IT department…but this technology is so far beyond where I was… I know it knows so many things, but I don’t know how to do them or get to them or where do I find a list of them? Alexa comes with…just a little card that says, download the app… but the app is more overwhelming…it’s not that easy.

A small piece of paper (which P13 refers to as “a little card”) is contributing to a situation where P13 experiences frustration and is overwhelmed to set up the technology. P8 describes how she misses having paper manuals: *“I think that’s the issue for me. I’m old school…give me information on paper, but there’s no manual for these [voice assistants]… And it makes me think, well what else am I missing out on.”* As such, absence of physical paper manuals (a nonhuman entity) contributes to a situation where it becomes harder for some older adults to learn about technology features.

### 3.3 Pets

A few participants’ accounts suggest their pet animal as a nonhuman actor in the socio-material configuration in which the voice assistant was used. P18 described a situation where his service dog followed Alexa’s command each day to go and fetch his medicine:

At 8:30, I had to take a shot and I’d blunder it all the time…I had set alarms on my phone, but then I would ignore it…that person there who’s talking [Alexa] would come on at 8:30 and she would announce that…[P18’s dog], she needs to go get my medication. The word was, “Go get it.” And so [P18’s dog], she’d run every day at 8:30…and go get it. It’s in a cupboard in the bathroom, the bottom cupboard, takes the satchel, brings it to me… and then I take my shot.

What P18 describes hints how the action of remembering when to take medication and how to access medicines involves nonhuman entities, including his service dog, the material arrangement and presence of medicine in the bathroom cabinet, as well as Alexa. In this situation, the dog or the cabinet, are no longer a passive entity in P18’s house. Instead, the intra-actions involving the pet animal, cupboard, and Alexa, is actively enacting the phenomenon of P18 taking his medication.

## 4 Discussion and Conclusion

Our findings illustrate cases of how nonhuman entities and material objects can shape technology use. While the role of nonhuman entities has been well noted in the broader technology and design context [7, 28], it remains under-discussed in context of technologies for aging. Below we discuss how accounting for nonhuman actors can help further understand technology non-use by older adults.

Understanding older adults’ non-use of technology is an area extensively studied. [3, 12, 29]. Research has identified different factors associated with non-use, including the social setting of use [24, 29], confidence associated with technology use [29], lack of perceived usefulness or utility [3, 15], the stigma that individuals may associate with some technologies [3, 18] or, the additional work/labor one has to perform to use/maintain technology [12, 14]. In this context, attuning to nonhuman entities can contribute towards a richer understanding of technology non-use as well as assign accountability to material actors rather than older adults for contributing towards this phenomenon. As an example, for decades paper manuals have been the primary actor in how older adults learn and remember how to use new technologies [25]. But emerging technologies are now disrupting these material configurations, where emails or phone apps are replacing paper manuals. By probing to identify such invisible material or nonhuman actors to understand who the actors are, and how they intra-act, we can account for them in our understanding, theories, design, processes, and decisions. We have found this sensitization helpful in our own work [9, 19], by closely paying attention to things that we previously did not take into account, such as instruction manuals, organization of space, position and mobility of furniture and technology, placement of outlets in walls, or the role of materials in research sessions. For example, in our ongoing projects involving setting up a new technology environment in a retirement community [9], we co-designed technology instruction manuals with residents.

Given the active role that nonhumans can play, we argue for holding these actors responsible and accountable when necessary. Considering the example of user manuals above, one might question: what difference does it make to say, ‘older adults experience barriers in using voice assistants due to difficulty pairing smartphones or remembering voice commands [20], vs. saying ‘missing material actors such as onboarding paper manuals create barriers in learning to use technology’? By re-framing responsibility and accountability to material actors, we can distance from the deficit model around the decline associated with aging and older adults being less tech-savvy or not being able to use technologies. Repeatedly assuming that older adults are less tech-savvy and experience challenges in using technology might not solve the issues around usability and perceived usefulness until we hold the actual material (in addition to social) actors responsible for any undesired situation. To achieve this, we need to expand our current ways of doing research to additionally think about what actions, we, as designers and researchers, can take to enable participation of nonhuman entities in our design process, such as by leveraging the concept of *repertoires* to make nonhumans more present and participatory through designers [28], thinking or speaking on behalf of nonhuman entities to probe an understanding of the larger socio-material network [4], or adopt speculative design approaches [21].

## CCS Concepts

• Human-centered computing → Human computer interaction (HCI); • Social and professional topics → Seniors.

## Footnotes

1 Adopting a new ontological framework, the posthuman turn in aging studies acknowledges the active participation of nonhuman entities—both biological and material—as agents in shaping the realities and experiences of aging. This perspective treats human and nonhuman entities as ontologically equivalent, recognizing that all possess agency, have the capacity for co-evolution [1]. In line with this ontological and epistemological perspective, we refer to nonhuman objects or biological entities as actors or entities.
